# Crystal structure and Hirshfeld surface analysis of 2,6-di­iodo-4-nitro­toluene and 2,4,6-tri­bromo­toluene

**DOI:** 10.1107/S2056989020010270

**Published:** 2020-07-31

**Authors:** Mohamed Larbi Medjroubi, Ali Boudjada, Noudjoud Hamdouni, Ouarda Brihi, Olivier Jeannin, Jean Meinnel

**Affiliations:** a Laboratoire de Cristallographie, Département de Physique, Université Mentouri-Constantine, 25000 Constantine, Algeria; b UMR 6226 CNRS–Université Rennes 1 ‘Sciences Chimiques de Rennes’, Equipe ‘Matière Condensée et Systèmes Electroactifs’, Bâtiment 10C Campus de Beaulieu, 263 Avenue du Général Leclerc, F-35042 Rennes, France

**Keywords:** X-ray diffraction, crystal structure, 2,6-di­iodo-4-nitro­toluene, 2,4,6-tri­bromo­toluene, Hirshfeld surface

## Abstract

The title compounds, 2,6-di­iodo-4-nitro­toluene (DINT, C_7_H_5_I_2_NO_2_) and 2,4,6-tri­bromo­toluene (TBT, C_7_H_5_Br_3_,), are tris­ubstituted toluene mol­ecules. Both mol­ecules are planar, only the H atoms of the methyl group, and the nitro group in DINT, deviate significantly from the plane of the benzene ring. In the crystals of both compounds, mol­ecules stack in columns up the shortest crystallographic axis, *viz*. the *a* axis in DINT and the *b* axis in TBT. In the crystal of DINT, mol­ecules are linked *via* short N—O⋯I contacts, forming chains along [100]. In TBT, mol­ecules are linked by C—H⋯Br hydrogen bonds, forming chains along [010]. Hirshfeld surface analysis was used to explore the inter­molecular contacts in the crystals of both DINT and TBT.

## Abstract 2

The crystal structures of DIN and TBT were determined by X-ray diffraction at 150 K. In the crystal of DINT, mol­ecules are linked *via* short N—O⋯I contacts, forming chains along [100]. In TBT, mol­ecules are linked by C—H⋯Br hydrogen bonds, forming chains along [010].

## Chemical context   

In order to understand the methyl radical behaviour of benzene mol­ecules substituted by halogens and methyl groups, we have studied a number of halogenomesitylenes, such as tri­iodo­mesitylene (TIM; Boudjada *et al.*, 2001 ▸), tri­chloro­mesitylene (TCM; Tazi *et al.*, 1995 ▸), tri­bromo­mesitylene (TBM; Boudjada *et al.*, 1999 ▸) and di­bromo­mesitylene (DBM; Hernandez *et al.*, 2003 ▸). In the solid state of these halogeno-methyl-benzene (HMB) compounds, the steric hindrance between the methyl group and the halogen atoms results in small out-of -plane deformations of the heavy atoms. In spite of the small amplitudes of the deformation, the impact on the rotational potential of the methyl group is considerable because of the contribution of the neighbouring halogen atoms on the methyl groups as observed in these planar structures. This study has now been extended to understand and identify the methyl-group behaviour of halogeno-toluene mol­ecules, and we report herein on the crystal and mol­ecular structures of the title compounds, 2,6-di­iodo-4-nitro­toluene (DINT; systematic name: 1,3-di­iodo-2-methyl-5-nitro­benzene) and 2,4,6-tri­bromo­toluene (TBT; systematic name: 1,3,5-tri­bromo-2-methyl-benzene). Hirshfeld surface analysis was used to explore the inter­molecular contacts in the crystals of both compounds.

## Structural commentary   

**Figure 1:**
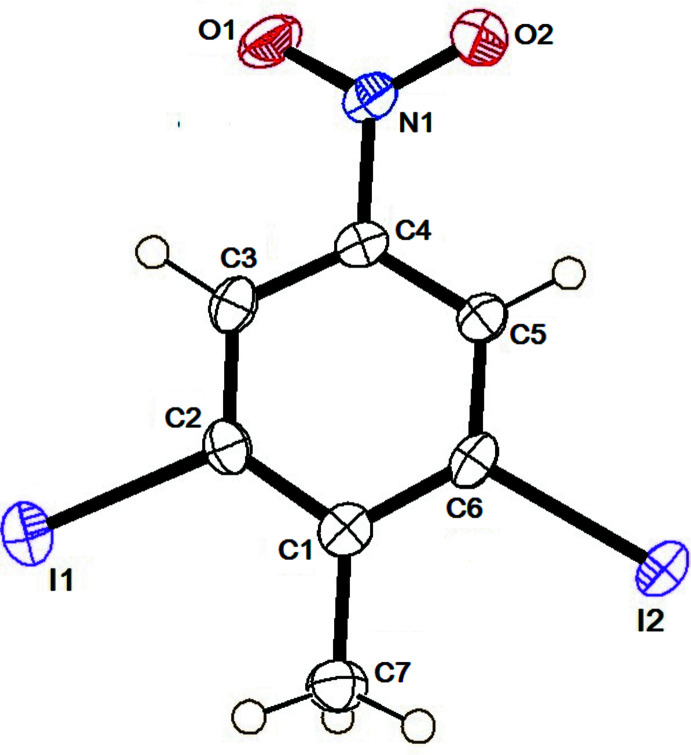
The mol­ecular structure of DINT with the atom labelling and displacement ellipsoids drawn at the 50% probability level.

**Figure 2:**
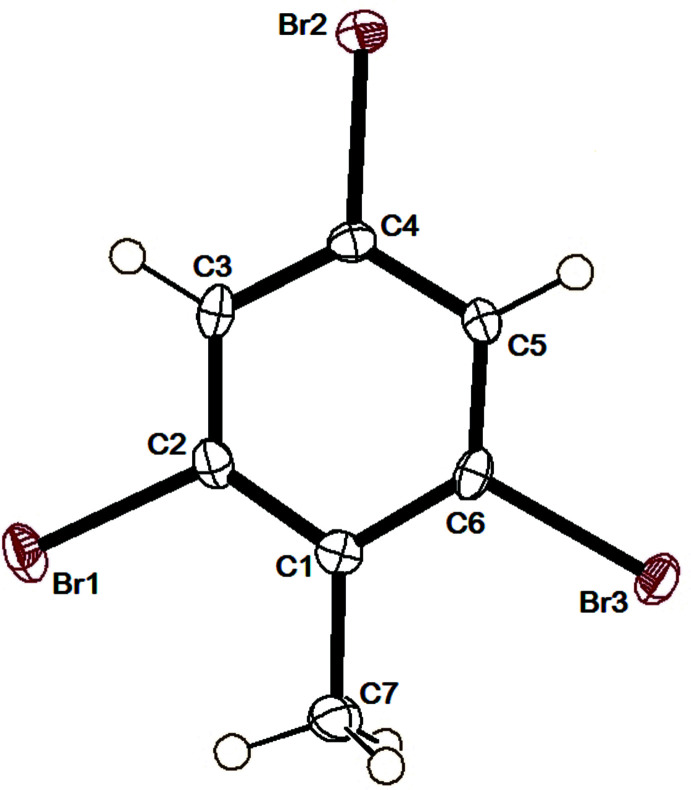
The mol­ecular structure of TBT with the atom labelling and displacement ellipsoids drawn at the 50% probability level.

**Figure 3:**
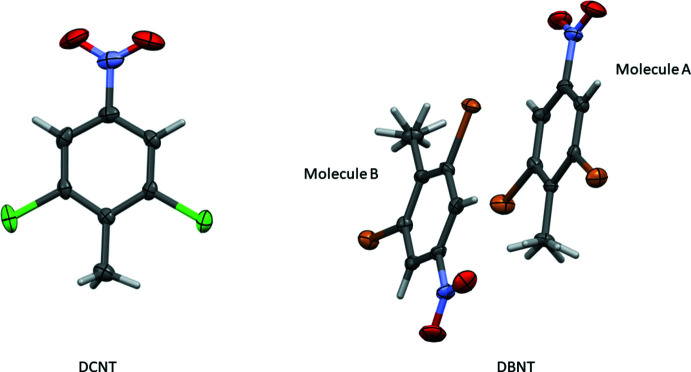
The mol­ecular structures of DCNT and DBNT, with displacement ellipsoids drawn at the 50% probability level.

The mol­ecular structure of DINT is illustrated in Fig. 1 ▸, and that of TBT in Fig. 2 ▸. The structures of the title compounds are compared with those of the di­chloro­nitro­toluene (DCNT; Medjroubi *et al.*, 2017 ▸) and di­bromo­nitro­toluene (DBNT; Medjroubi *et al.*, 2016 ▸) analogues, illustrated in Fig. 3 ▸.
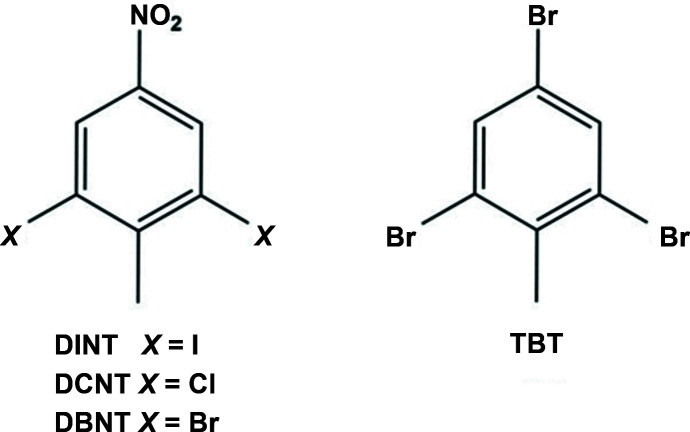


**Figure 4:**
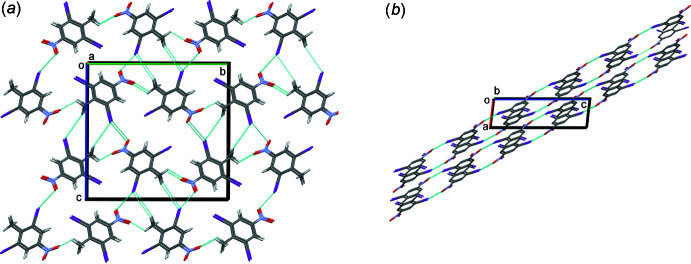
A view along the *a* and *b* axes of the crystal packing of DINT.

**Figure 5:**
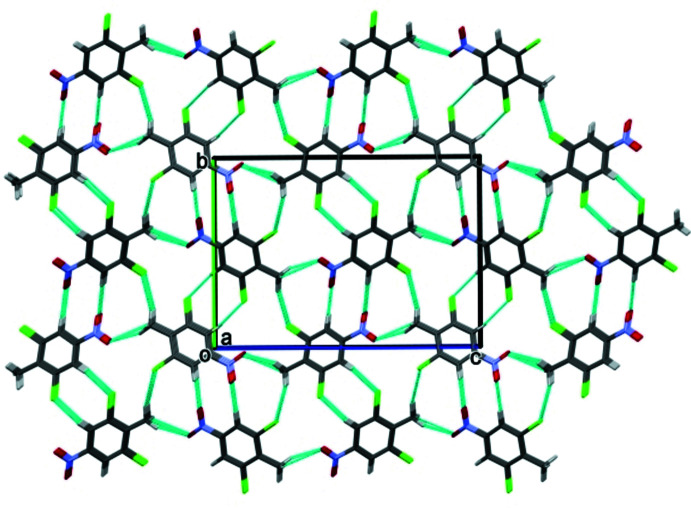
A view along the *a* axes of the crystal packing of 2,6-di­chloro-4-nitro­toluene, DCNT.

In DBNT, the methyl group exhibits rotational disorder about the C_ar_—C_me_ axis. As in DCNT, the methyl group of DINT does not present any disorder. Hence, no significant steric hindrance of the methyl group by the *ortho* halogen atoms is observed. The longest bond lengths C_ar_—C_ar_ are adjacent to the C_ar_—C_me_ bond with an average value of 1.405 (4) Å. The bond-length values are close to those found in the literature. Moreover, in the crystal structure of DINT, the methyl group has a C—H bond perpendicular to the mean plane with a torsion angle C2—C1—C7—H7*A* = 90.0°, which has already previously been reported in the literature. The inter­molecular N1—O⋯I [3.115 (3) Å] inter­actions are competing to ensure cohesion in the crystal, see Fig. 4 ▸. Atom I2 bonded to the atom C6, with the C1—C6—I2 angle of 121.7 (3)° being greater than the angle C5—C6—I2 [116.1 (2)°] located on the other side of the C6—I2 bond (Fig. 1 ▸). The aromatic ring is planar in DINT. The methyl C atom, the I atoms and the N atom of the nitro substituent, lie extremely close to the plane of the benzene ring; the deviations are 0.001 (1) Å for the methyl C atom, 0.097 (3) and −0.097 (3) Å for the two I atoms, −0.011 (2) Å for the nitro N atom, whereas for the oxygen atoms, which are located on either side of the mean plane, the deviations are 0.293 (3) and −0.283 (3) Å. In the crystal of DCNT, mol­ecules are linked by weak C—H⋯O and C—H⋯Cl hydrogen bonds, forming layers parallel to the *ab* plane Fig. 5 ▸ (Medjroubi *et al.*, 2017 ▸).

**Figure 6:**
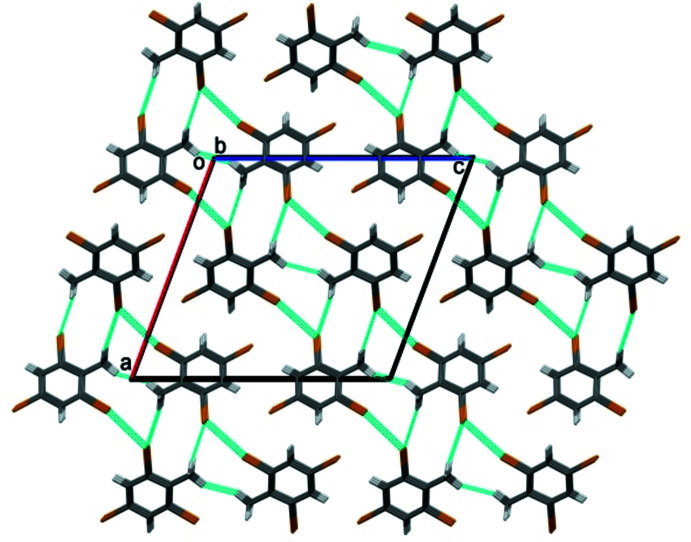
A view along the *b* axes of the crystal packing of 2,4,6-tri­bromo­toluene, DBNT.

The mol­ecular structure of TBT is illustrated in Fig. 2 ▸. The structural study did not reveal any disorder and the intra­molecular inter­action ensuring the cohesion in the crystal is C—Br⋯ H7*B* (2.61 Å) as seen in Fig. 6 ▸ and Table 1 ▸. This conformation produces a significant steric effect between the hydrogen atoms of the methyl group and atom Br2 bonded to atom C6 with an angle C1—C6—Br3 = 119.8 (2)°, which is clearly greater than the angle C5—C6—Br3 = 116.5 (2)° located on the other side of the C6—Br1 bond. This is the same case for the exocyclic angles C6—C1—C7 = 121.9 (3)° and C2—C1—C7 = 123.6 (3)° located on either side of the C1—C7 bond. As found in DINT and DCNT, the longest C_ar_—C_ar_ bond lengths are adjacent to the C_ar_–C_me_ bond (Fig. 2 ▸). The methyl C atom C7 is displaced from the benzene ring by −0.005 (1) Å, while the Br atoms lie almost in the plane of the benzene ring [deviations are 0.003 (3) Å for atoms Br1 and Br3 and −0.012 (3)  Å for atom Br2]. The CH_3_ group presents an eclipsed C—H bond with the mean plane of the mol­ecule. A difference of 2° is found between the exocyclic angles C_ar_— C_ar_—C_me_, which explains the importance of the inter­action of this eclipsed bond with atom Br3. The endocyclic angle in front of the methyl group is equal to 116.6 (3)° for DINT, 115.7 (2)° for DCNT (Medjroubi *et al.*, 2017 ▸), 114.7 (3)° for DBNT (Medjroubi *et al.*, 2016 ▸) and 114.5 (3)° for TBT. The variation of this angle is very sensitive to the substitution of the halogen atoms surrounding the methyl groups of these different compounds.

## Supra­molecular features   

In the crystal of DINT, the mol­ecules are assembled into columns along the *a*-axis direction, the shortest crystallographic axis. Mol­ecules are linked by O⋯I inter­molecular inter­actions, with distance I2⋯O1^i^ = 3.12 (1) Å [symmetry code (i): *x* − 1, −*y* + 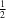, *z* + 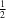], leading to the formation of chains along the [20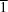] direction, see Fig. 4 ▸.

In the crystal of TBT, mol­ecules stack in columns along the *b*-axis direction, again the shortest crystallographic axis. Mol­ecules are linked by weak Br⋯Br inter­actions [Br1⋯Br3^ii^ = 3.5921 (5) Å; symmetry code (ii): *x* + 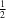, −*y* + 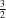, *z* + 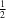], forming chains along the [101] direction. Br⋯H short contacts are also present in the crystal of TBT [Br1⋯H7*C* ^ii^ = 3.5921 (5) Å; symmetry code (iii): *x*, −1 + *y*, *z*].

## Analysis of the Hirshfeld surfaces of DINT and TBT   

**Figure 7:**
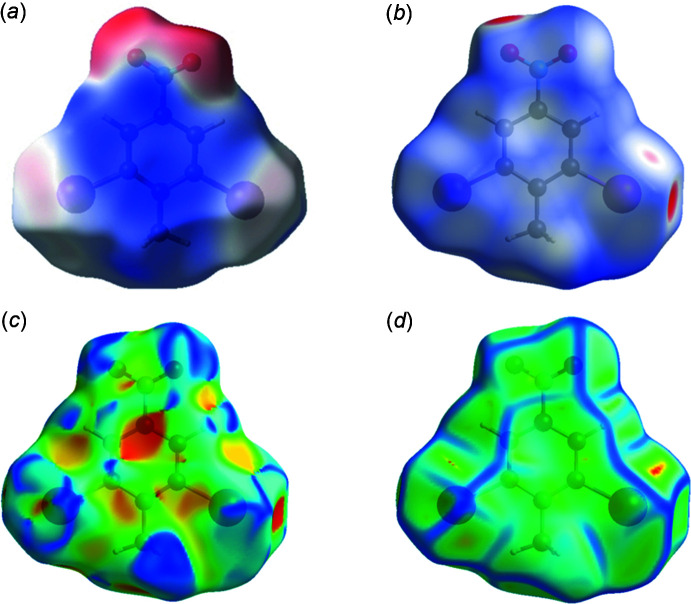
Hirshfeld surface for DINT mapped over (*a*) calculated electrostatic potential, (*b*) *d*_norm_, (*c*) shape-index and (*d*) curvedness.

Additional insight into the inter­molecular inter­actions was obtained from analysis of the Hirshfeld surface (HS) (Spackman & Jayatilaka, 2009 ▸) and the two-dimensional fingerprint plots (McKinnon *et al.*, 2007 ▸). The program *CrystalExplorer* (Turner *et al.*, 2017 ▸) was used to generate Hirshfeld surfaces mapped over *d*_norm_ and the electrostatic potential for compounds TBT and DINT. The function *d*_norm_ is a ratio enclosing the distances of any surface point to the nearest interior (*d*_i_) and exterior (*d*_e_) atom and the van der Waals (vdW) radii of the atoms. The electrostatic potentials were calculated using *TONTO* (Spackman & Jayatilaka, 2009 ▸) integrated into *CrystalExplorer*, using the crystal structure as the starting geometry. Short contacts and contributions to the Hirshfeld surface for DINT, DCNT (Medjroubi *et al.*, 2017 ▸) and TBT are given in Table 2 ▸. The Hirshfeld surface (HS) mapped over the electrostatic potential for DINT in the range [−0.071 to +0.041], is shown in Fig. 7 ▸ *a* where the red and blue regions represent negative and positive electrostatic potentials respectively. The Hirshfeld surface mapped over *d_norm_* is depicted in Fig. 7 ▸ *b*. The HS mapped over shape-index and curvedness are shown in Fig. 7 ▸ *c* and 7*d*, respectively.

**Figure 8:**
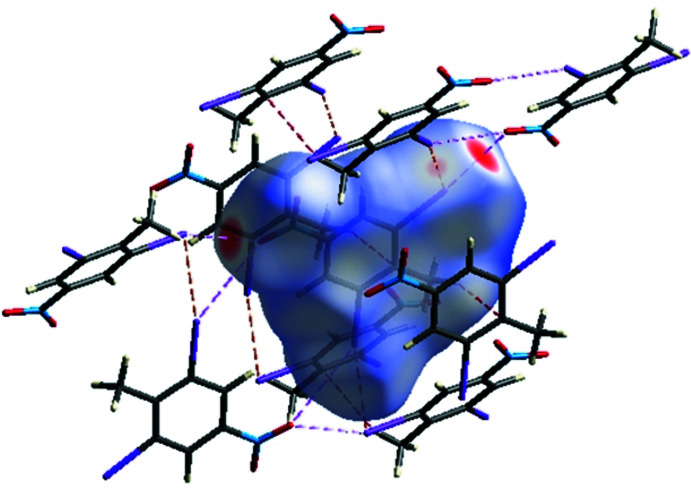
A view of the Hirshfeld surface of DINT mapped over *d*_norm_, with inter­actions shown as dashed lines.

**Figure 9:**
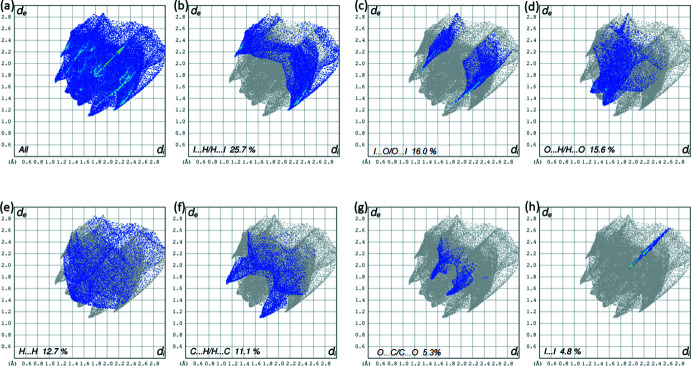
(*a*) The full two-dimensional fingerprint plot calculated for DINT and those delineated into (*b*) I⋯H/H⋯I contacts, (*c*) I⋯O/O⋯I contacts, (*d*) O⋯H/H⋯O contacts, (*e*) H⋯H contacts, (*f*) C⋯H/H⋯C contacts, (*g*) O⋯C/C⋯O contacts and (*h*) I⋯I contacts.

**Figure 10:**
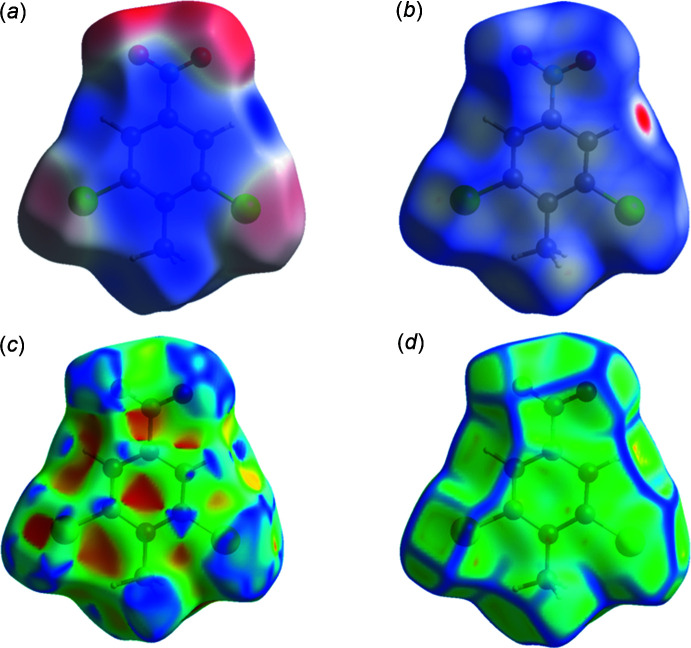
Hirshfeld surface for DCNT mapped over (*a*) calculated electrostatic potential, (*b*) *d*_norm_, (*c*) shape-index and (*d*) curvedness.

**Figure 11:**
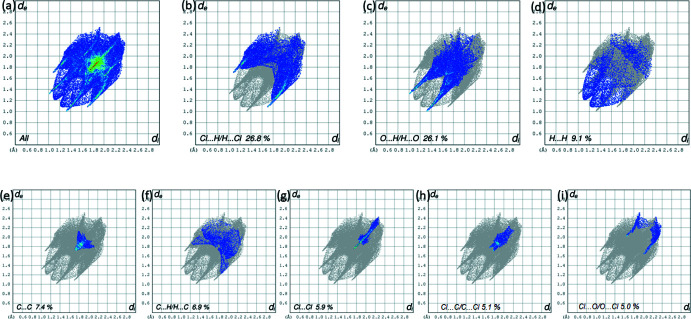
(*a*) The full two-dimensional fingerprint plot calculated for DCNT and those delineated into (*b*) Cl⋯H/H⋯Cl contacts, (*c*) O⋯H/H⋯O contacts, (*d*) H⋯H contacts, (*e*) C⋯C contacts, (*f*) C⋯H/H⋯C contacts, (*g*) Cl⋯Cl contacts, (*h*) Cl⋯C/C⋯Cl contacts and (*i*) Cl⋯O/O⋯Cl contacts.

The crystal environment about a DINT mol­ecule is illus­trated in Fig. 8 ▸: the inter­actions are shown on the Hirshfeld surfaces with short contacts indicated in red. The two-dimensional fingerprint plots for all contacts are illustrated in Fig. 9 ▸ *a*. The I⋯O/O⋯I inter­action ensures the cohesion of the crystal with a contribution of 16% of all the inter­actions appearing in bright-red spots on the Hirshfeld surfaces mapped over *d*_norm_. The fingerprint consists of two spikes with the ends at *d*_e_ + *d*_i_ ≃ 3.1 Å, Fig. 9 ▸ *c*. The fingerprint of the C⋯H/H⋯C inter­action consists of two symmetrical peaks with a *d*_e_ + *d*_i_ ≃ 2.8 Å, Fig. 9 ▸ *f* and almost equal to the sum of van der Waals radii. It is represented on the HS mapped with the shape-index property in the form of a pale-red spot, Fig. 7 ▸ *c*. The I⋯I inter­action is less important than O⋯I/I⋯O; however, it does contribute 4.8% to the total inter­actions, illustrating the equality of the I⋯I distance to the sum of van der Waals radii. The fingerprint is composed of a single peak in the form of a needle with *d*_e_ + *d*_i_ ≃ 3.8 Å, Fig. 9 ▸ *h*. The absence of π–π stacking inter­actions is consistent with the low contributions of C⋯C contacts to the Hirshfeld surface (Table 2 ▸). The Hirshfeld surface analysis (Fig. 10 ▸ *b*) and electrostatic potential surface (Fig. 10 ▸ *a*) show the inter­molecular inter­actions between different units in the crystalline environment of DCNT. The HS mapped over shape-index and curvedness are shown in Fig. 10 ▸ *c* and *d*, respectively. The two-dimensional fingerprint plots for all contacts are illustrated in Fig. 11 ▸. The contributions of the major inter­molecular contacts in the title compound are Cl⋯H/H⋯Cl (26.8%), O⋯H/H⋯O (26.1%), and H⋯H (10.6%) (Fig. 11 ▸ *a*). Other contacts (*e.g.* C⋯C, H⋯C/C⋯H, Cl⋯Cl, Cl⋯C/C⋯Cl, Cl⋯O/O⋯Cl) make contributions of less than 7.5% to the HS. The graph shown in Fig. 11 ▸ *b* represents the one-third of all the inter­molecular contacts. All fingerprint points are located at distances with *d*_i_ equal to or greater than van der Waals distances, *d*_e_ + *d*_i_ ≃ 1.27 Å, reflecting a zero tendency to form this inter­molecular contact (Cl⋯H). The graph shown in Fig. 11 ▸ *c* (O⋯H/H⋯O) shows the contact between the oxygen atoms inside the surface and the hydrogen atoms outside the surface, *d*_e_ + *d*_i_ ≃ 2.35 Å, and has two symmetrical points at the top, bottom left and right. The graph shown in Fig. 11 ▸ *d* (H⋯H; 10.6%) shows the two-dimensional fingerprint of the (*d*_i_, *d*_e_) points associated with hydrogen atoms, which has two symmetrical wings on the left and right with *d*_e_ + *d*_i_ ≃ 2.3 Å.

**Figure 12:**
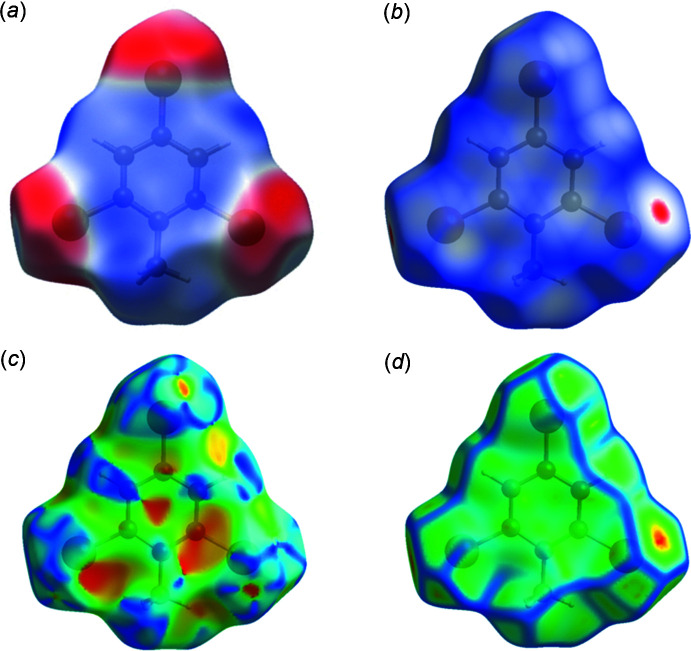
Hirshfeld surface for TBT mapped over (*a*) calculated electrostatic potential, (*b*) *d*_norm_, (*c*) shape-index and (*d*) curvedness.

**Figure 13:**
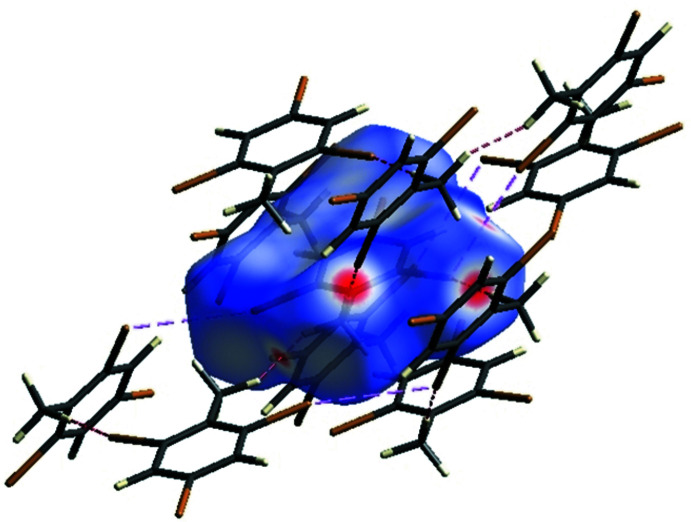
A view of the Hirshfeld surface of TBT mapped over *d*_norm_, with inter­actions shown as dashed lines.

**Figure 14:**
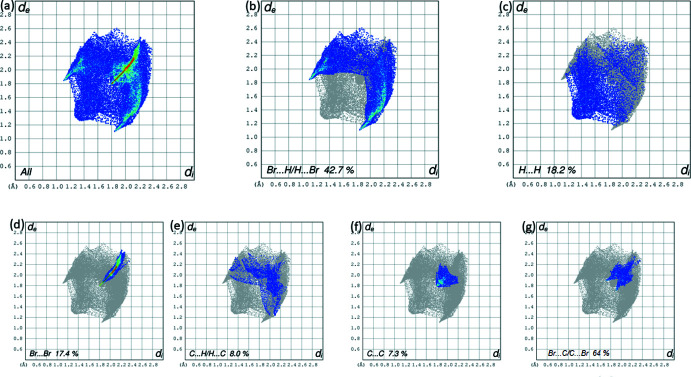
(*a*) The full two-dimensional fingerprint plot calculated for TBT and those delineated into (*b*) Br⋯H/H⋯Br contacts, (*c*) H⋯H contacts, (*d*) Br⋯Br contacts, (*e*) C⋯H/H⋯C contacts, (*f*) C⋯C contacts and (*g*) Br⋯C/C⋯Br contacts.

The electrostatic potentials were mapped on the Hirshfeld surface using the STO-3G basis/Hartree–Fock level of theory over the range [−0.016, 0.036] for TBT (Fig. 12 ▸). Atoms H7*A*, H7*B* and H7*C* of the methyl group and H3, H5 as donors, and the bromine atom acceptors, are also evident in Fig. 12 ▸ *a*. The Hirshfeld surface mapped over *d_norm_* is depicted in Fig. 12 ▸ *b*. The overall 2D fingerprint plot is presented in Fig. 13 ▸ *a*. The halogen–halogen (Br⋯Br) inter­action contributes 17.4% to the HS and ensures the cohesion of the crystal and dictates the inter­molecular stacking. The short intra­molecular H⋯Br/Br⋯H contact between C3—H7*B* and Br1 (H7*B*⋯Br1 = 2.6 Å) can be recognized from the two neighbouring blue regions on the surface mapped with electrostatic potential in Fig. 14 ▸ *c*,*d*. The two-dimensional fingerprint plot delineated into Br⋯H/H⋯Br has two peaks pointing to the pairs *d*_e_ + *d*_i_ ≃ 3.0 Å, slightly lower than or equal to the sum of van der Waals radii, Fig. 14 ▸ *b*. These correspond to a 42.7% contribution to the Hirshfeld surface, and reflect the presence of inter­molecular C7—H7*C*⋯Br3 inter­actions. The inter­atomic H⋯H contacts at distances greater than their van der Waals separation appear as scattered points in the greater part of the fingerprint plot Fig. 14 ▸ *c*. The presence of C—H⋯π and H—C⋯π [or π–π ?]stacking inter­actions between the TBT rings is also apparent from the appearance of red and blue triangle pairs on the Hirshfeld surface mapped with shape-index property identified with arrows in Fig. 12 ▸ *c*. The immediate environment about each mol­ecule highlighting close contacts to the Hirshfeld surface by neighboring mol­ecules is shown in Fig. 13 ▸. The relative contributions to the overall surface are given in Table 2 ▸.

## Database survey   

A search of the Cambridge Structural Database (CSD, Version 5.40, last update May 2019; Groom *et al.*, 2016 ▸) for 2,6-di­iodo-4-nitro­toluene (DINT) and 2,4,6-tri­bromo­toluene (TBT) gave five hits: 2,6-di­chloro-4-nitro­toluene (DCNT; Medjroubi *et al.*, 2017 ▸), di­bromo­nitro­toluene (DBNT; Medjroubi *et al.*, 2016 ▸), tri­iodo­mesitylene (TIM) (Boudjada *et al.*, 2001 ▸), tri­bromo­mesitylene (TBM; Boudjada *et al.*, 1999 ▸) and di­bromo­mesitylene (DBM; Hernandez *et al.*, 2003 ▸). In DBNT (Medjroubi *et al.*, 2016 ▸), there are two independent mol­ecules per asymmetric unit and the methyl group H atoms are positionally disordered, as found for DBM (Hernandez *et al.*, 2003 ▸). While in the compounds DINT and DCNT (Medjroubi *et al.*, 2017 ▸), there is only one mol­ecule in the asymmetric unit and no disorder is observed for the methyl group H atoms. In the mol­ecule of DINT, a C_m_—H (m = meth­yl) bond is perpendicular to the mean plane of the mol­ecule, as found for tri­iodo­mesitylene (TIM; Boudjada *et al.*, 2001 ▸). The nitro group is inclined to the benzene ring by 16.72 (1)° in DINT, compared with 9.8 (3)° in DCNT, and 2.5 (5)° and 5.9 (4)° in DBNT. In the mol­ecule of TBT, the CH_3_ group presents an eclipsed C—H bond with the mean plane of the mol­ecule. This also applies to DCNT (Medjroubi *et al.*, 2017 ▸), which does not present any disorder, as was also found in the case of tri­bromo­mesitylene TBM (Boudjada *et al.*, 1999 ▸). In 2,6-dihalogeno-4-nitro­toluene, as in the title compounds, the cohesion of the crystal is ensured by inter­actions of the type C—H⋯halogen and C—halogen⋯halogen.

## Synthesis and crystallization   

**2,6-Di­iodo-4-nitro­toluene (DINT):** 4-nitro­toluene (0.68g, 5 mmol) was suspended in 90% (*v*/*v*) conc. H_2_SO_4_ (10 ml) at 298–303 K. While keeping the same temperature, the iodinating solution containing the I^+^ inter­mediate in *ca* 50% excess was added dropwise under stirring over 45 min. The stirring was continued at 298–303 K for a further 75 min. The final reaction mixture was poured, with stirring, into ice–water (300 g). The crude solid obtained was recrystallized from ethanol (27 ml). On slow evaporation of the solvent, colourless prismatic crystals of DINT were obtained (yield 77%, 1.5 g).

**2,4,6-Tri­bromo­toluene (TBT)** is commercially available (Sigma–Aldrich). It was recrystallized from ethanol solution, giving large colourless needle-like crystals, many of which were twinned.

## Refinement details   

Crystal data, data collection and structure refinement details for DINT and TBT are summarized in Table 3 ▸. The H atoms were included in calculated positions and refined as riding: C—H = 0.95–0.98 Å with *U*_iso_(H) = 1.5*U*_eq_(C-meth­yl) and 1.2*U*_eq_(C) for other H atoms.

## Supplementary Material

Crystal structure: contains datablock(s) global, DINT, TBT. DOI: 10.1107/S2056989020010270/dj2011sup1.cif

Structure factors: contains datablock(s) DINT. DOI: 10.1107/S2056989020010270/dj2011DINTsup2.hkl

Structure factors: contains datablock(s) TBT. DOI: 10.1107/S2056989020010270/dj2011TBTsup3.hkl

CCDC references: 2018692, 2018691

Additional supporting information:  crystallographic information; 3D view; checkCIF report

## Figures and Tables

**Table 1:** Hydrogen bonds (Å, °) for (I) and (II)

Compound	*D*—H⋯*A*	*D*—H	H⋯*A*	*D*⋯*A*	*D*—H⋯*A*
(I)	C7—H7*B*⋯I1	0.98	2.82	3.333 (4)	113
	C7—H7*C*⋯I2	0.98	2.84	3.331 (4)	112
(II)	C7—H7*B*⋯Br1	0.98	2.61	3.199 (3)	118

**Table 2:** Short contacts and contributions (%) to the Hirshfeld surface for DINT, DCNT and TBT

DINT		DCNT		TBT	
Contact	%	Contact	%	Contact	%
I⋯H/H⋯I	25.7	Cl⋯H/H⋯Cl	26.8	Br⋯H/H⋯Br	42.7
I⋯O/O⋯I	16.0				
O⋯H/H⋯O	15.6	O⋯H/H⋯O	26.1		
H⋯H	12.7	H⋯H	9.1	H⋯H	18.2
C⋯H/H⋯C	11.1	C⋯H/H⋯C	6.9	C⋯H/H⋯C	8.0
O⋯C/C⋯O	5.3				
I⋯I	4.8	Cl⋯Cl	5.9	Br⋯Br	17.4
C⋯C	3.4	C⋯C	7.4	C⋯C	7.3
		Cl⋯O/O⋯Cl	5.0		
		Cl⋯C/C⋯Cl	5.1	Br⋯C/C⋯Br	6.4

**Table 3:** Experimental details

	DINT	TBT
Crystal data		
Chemical formula	C_7_H_5_I_2_NO_2_	C_7_H_5_Br_3_
*M*_r_	388.92	328.84
Crystal system, space group	Monoclinic, *P*2_1_/*c*	Monoclinic, *P*2_1_/*n*
Temperature (K)	150	150
*a*, *b*, *c* (Å)	4.3815 (2), 15.3348 (6), 14.5894 (6)	14.3484 (11), 3.9955 (3), 15.6975 (12)
β (°)	96.588 (1)	110.519 (2)
*V* (Å^3^)	973.78 (7)	842.83 (11)
*Z*	4	4
Radiation type	Mo *K*α	Mo *K*α
μ (mm^−1^)	6.42	14.28
Crystal size (mm)	0.29 × 0.13 × 0.06	0.34 × 0.21 × 0.12

Data collection		
Diffractometer	Bruker APEXII	Bruker APEXII
Absorption correction	Multi-scan (*SADABS*; Bruker, 2006 ▸)	Multi-scan (*SADABS*; Bruker, 2006 ▸)
*T*_min_, *T*_max_	0.382, 0.680	0.496, 0.746
No. of measured, independent and observed [*I* > 2σ(*I*)] reflections	4104, 2244, 1850	6197, 1928, 1657
*R*_int_	0.022	0.027
(sin θ/λ)_max_ (Å^−1^)	0.651	0.650

Refinement		
*R*[*F* ^2^ > 2σ(*F* ^2^)], *wR*(*F* ^2^), *S*	0.024, 0.046, 0.98	0.025, 0.049, 1.08
No. of reflections	2244	1928
No. of parameters	110	93
H-atom treatment	H-atom parameters constrained	H-atom parameters constrained
Δρ_max_, Δρ_min_ (e Å^−3^)	0.65, −0.55	0.70, −0.68

Computer programs: *APEX2* and *SAINT* (Bruker, 2006 ▸), *SIR2004* (Burla *et al.*, 2005 ▸), *SHELXL2018/3* (Sheldrick, 2015 ▸), *ORTEP-3 for Windows* and *WinGX* publication routines (Farrugia, 2012 ▸) and *Mercury* (Macrae *et al.*, 2020 ▸).

## supplementary crystallographic information

### 2,6-Diiodo-4-nitrotoluene (DINT) Crystal data

**Table:**

C_7_H_5_I_2_NO_2_	*F*(000) = 704
*M**_r_* = 388.92	*D*_x_ = 2.653 Mg m^−^^3^
Monoclinic, *P*2_1_/*c*	Mo *K*α radiation, λ = 0.71073 Å
Hall symbol: -P 2ybc	Cell parameters from 3615 reflections
*a* = 4.3815 (2) Å	θ = 2.8–27.4°
*b* = 15.3348 (6) Å	µ = 6.42 mm^−^^1^
*c* = 14.5894 (6) Å	*T* = 150 K
β = 96.588 (1)°	Prism, colourless
*V* = 973.78 (7) Å^3^	0.29 × 0.13 × 0.06 mm
*Z* = 4	

### 2,6-Diiodo-4-nitrotoluene (DINT) Data collection

**Table:**

Bruker APEXII diffractometer	1850 reflections with *I* > 2σ(*I*)
CCD rotation images, thin slices scans	*R*_int_ = 0.022
Absorption correction: multi-scan (SADABS; Bruker, 2006)	θ_max_ = 27.5°, θ_min_ = 2.7°
*T*_min_ = 0.382, *T*_max_ = 0.680	*h* = −5→5
4104 measured reflections	*k* = −19→19
2244 independent reflections	*l* = 0→18

### 2,6-Diiodo-4-nitrotoluene (DINT) Refinement

**Table:**

Refinement on *F*^2^	Primary atom site location: structure-invariant direct methods
Least-squares matrix: full	Secondary atom site location: difference Fourier map
*R*[*F*^2^ > 2σ(*F*^2^)] = 0.024	Hydrogen site location: inferred from neighbouring sites
*wR*(*F*^2^) = 0.046	H-atom parameters constrained
*S* = 0.98	*w* = 1/[σ^2^(*F*_o_^2^) + (0.0064*P*)^2^] where *P* = (*F*_o_^2^ + 2*F*_c_^2^)/3
2244 reflections	(Δ/σ)_max_ = 0.001
110 parameters	Δρ_max_ = 0.65 e Å^−^^3^
0 restraints	Δρ_min_ = −0.55 e Å^−^^3^

### 2,6-Diiodo-4-nitrotoluene (DINT) Fractional atomic coordinates and isotropic or equivalent isotropic displacement parameters (Å^2^)

**Table:**

	*x*	*y*	*z*	*U*_iso_*/*U*_eq_	
I2	0.49927 (5)	0.16308 (2)	0.43507 (2)	0.02865 (8)	
I1	0.61809 (6)	−0.09049 (2)	0.11508 (2)	0.03239 (8)	
O2	1.0610 (7)	0.31383 (17)	0.16120 (19)	0.0439 (7)	
O1	1.2265 (6)	0.21412 (17)	0.07502 (18)	0.0383 (6)	
N1	1.0741 (6)	0.2380 (2)	0.1357 (2)	0.0270 (7)	
C4	0.9021 (7)	0.1719 (2)	0.1809 (2)	0.0213 (7)	
C5	0.7967 (7)	0.1926 (2)	0.2637 (2)	0.0226 (7)	
C7	0.4089 (8)	−0.0220 (2)	0.3155 (3)	0.0311 (8)	
C3	0.8521 (7)	0.0917 (2)	0.1389 (2)	0.0248 (8)	
C6	0.6400 (7)	0.1288 (2)	0.3069 (2)	0.0223 (7)	
C1	0.5769 (7)	0.0462 (2)	0.2676 (2)	0.0238 (8)	
C2	0.6870 (7)	0.0302 (2)	0.1830 (2)	0.0243 (8)	
H5	0.830322	0.248772	0.290300	0.027*	
H3	0.927896	0.078917	0.081954	0.030*	
H7A	0.556153	−0.056139	0.356537	0.047*	
H7B	0.298926	−0.060732	0.269445	0.047*	
H7C	0.261494	0.006076	0.351681	0.047*	

### 2,6-Diiodo-4-nitrotoluene (DINT) Atomic displacement parameters (Å^2^)

**Table:**

	*U*^11^	*U*^22^	*U*^33^	*U*^12^	*U*^13^	*U*^23^
I2	0.02982 (13)	0.03671 (15)	0.02075 (13)	0.00201 (11)	0.00862 (10)	−0.00039 (10)
I1	0.03450 (14)	0.02883 (14)	0.03341 (16)	−0.00032 (11)	0.00206 (11)	−0.00721 (11)
O2	0.0607 (19)	0.0330 (15)	0.0414 (18)	−0.0150 (14)	0.0212 (15)	−0.0067 (13)
O1	0.0426 (16)	0.0438 (16)	0.0318 (16)	0.0010 (13)	0.0189 (13)	0.0059 (13)
N1	0.0268 (15)	0.0334 (17)	0.0210 (16)	−0.0023 (14)	0.0039 (13)	0.0026 (14)
C4	0.0170 (16)	0.0263 (18)	0.0206 (18)	0.0018 (14)	0.0014 (13)	0.0045 (14)
C5	0.0216 (17)	0.0259 (18)	0.0200 (18)	0.0007 (14)	0.0009 (14)	0.0011 (15)
C7	0.0290 (19)	0.030 (2)	0.035 (2)	−0.0010 (17)	0.0083 (16)	0.0031 (17)
C3	0.0226 (17)	0.0310 (19)	0.0204 (19)	0.0045 (15)	0.0010 (14)	−0.0018 (15)
C6	0.0185 (16)	0.0330 (19)	0.0148 (18)	0.0066 (15)	−0.0007 (14)	0.0009 (15)
C1	0.0200 (16)	0.0279 (19)	0.0229 (19)	0.0055 (15)	−0.0003 (14)	0.0034 (15)
C2	0.0224 (17)	0.0233 (18)	0.026 (2)	0.0011 (15)	−0.0015 (15)	−0.0029 (15)

### 2,6-Diiodo-4-nitrotoluene (DINT) Geometric parameters (Å, º)

**Table:**

I2—C6	2.101 (3)	C7—C1	1.497 (5)
I1—C2	2.105 (3)	C7—H7A	0.9800
O2—N1	1.224 (4)	C7—H7B	0.9800
O1—N1	1.224 (4)	C7—H7C	0.9800
N1—C4	1.464 (4)	C3—C2	1.389 (5)
C4—C5	1.378 (5)	C3—H3	0.9500
C4—C3	1.381 (5)	C6—C1	1.404 (5)
C5—C6	1.388 (5)	C1—C2	1.398 (5)
C5—H5	0.9500		
			
O2—N1—O1	123.5 (3)	H7B—C7—H7C	109.5
O2—N1—C4	118.4 (3)	C4—C3—C2	117.6 (3)
O1—N1—C4	118.0 (3)	C4—C3—H3	121.2
C5—C4—C3	122.8 (3)	C2—C3—H3	121.2
C5—C4—N1	118.5 (3)	C5—C6—C1	122.3 (3)
C3—C4—N1	118.7 (3)	C5—C6—I2	116.1 (2)
C4—C5—C6	118.0 (3)	C1—C6—I2	121.7 (3)
C4—C5—H5	121.0	C2—C1—C6	116.5 (3)
C6—C5—H5	121.0	C2—C1—C7	121.8 (3)
C1—C7—H7A	109.5	C6—C1—C7	121.6 (3)
C1—C7—H7B	109.5	C3—C2—C1	122.7 (3)
H7A—C7—H7B	109.5	C3—C2—I1	115.7 (3)
C1—C7—H7C	109.5	C1—C2—I1	121.6 (3)
H7A—C7—H7C	109.5		
			
O2—N1—C4—C5	−15.8 (4)	C5—C6—C1—C2	1.5 (5)
O1—N1—C4—C5	163.4 (3)	I2—C6—C1—C2	−178.8 (2)
O2—N1—C4—C3	164.2 (3)	C5—C6—C1—C7	179.7 (3)
O1—N1—C4—C3	−16.7 (4)	I2—C6—C1—C7	−0.6 (4)
C3—C4—C5—C6	1.5 (5)	C4—C3—C2—C1	−1.2 (5)
N1—C4—C5—C6	−178.6 (3)	C4—C3—C2—I1	179.8 (2)
C5—C4—C3—C2	0.3 (5)	C6—C1—C2—C3	0.3 (5)
N1—C4—C3—C2	−179.7 (3)	C7—C1—C2—C3	−177.8 (3)
C4—C5—C6—C1	−2.4 (5)	C6—C1—C2—I1	179.2 (2)
C4—C5—C6—I2	177.9 (2)	C7—C1—C2—I1	1.1 (4)

### 2,4,6-Tribromotoluene (TBT) Crystal data

**Table:**

C_7_H_5_Br_3_	*F*(000) = 608
*M**_r_* = 328.84	*D*_x_ = 2.592 Mg m^−^^3^
Monoclinic, *P*2_1_/*n*	Mo *K*α radiation, λ = 0.71073 Å
*a* = 14.3484 (11) Å	Cell parameters from 2989 reflections
*b* = 3.9955 (3) Å	θ = 2.4–27.5°
*c* = 15.6975 (12) Å	µ = 14.28 mm^−^^1^
β = 110.519 (2)°	*T* = 150 K
*V* = 842.83 (11) Å^3^	Prism, colourless
*Z* = 4	0.34 × 0.21 × 0.12 mm

### 2,4,6-Tribromotoluene (TBT) Data collection

**Table:**

Bruker APEXII diffractometer	1657 reflections with *I* > 2σ(*I*)
CCD rotation images, thin slices scans	*R*_int_ = 0.027
Absorption correction: multi-scan (SADABS; Bruker, 2006)	θ_max_ = 27.5°, θ_min_ = 2.4°
*T*_min_ = 0.496, *T*_max_ = 0.746	*h* = −18→18
6197 measured reflections	*k* = −4→5
1928 independent reflections	*l* = −18→20

### 2,4,6-Tribromotoluene (TBT) Refinement

**Table:**

Refinement on *F*^2^	Secondary atom site location: difference Fourier map
Least-squares matrix: full	Hydrogen site location: inferred from neighbouring sites
*R*[*F*^2^ > 2σ(*F*^2^)] = 0.025	H-atom parameters constrained
*wR*(*F*^2^) = 0.049	*w* = 1/[σ^2^(*F*_o_^2^) + (0.0154*P*)^2^ + 1.1776*P*] where *P* = (*F*_o_^2^ + 2*F*_c_^2^)/3
*S* = 1.08	(Δ/σ)_max_ = 0.001
1928 reflections	Δρ_max_ = 0.70 e Å^−^^3^
93 parameters	Δρ_min_ = −0.68 e Å^−^^3^
0 restraints	Extinction correction: SHELXL-2018/3 (Sheldrick 2015), Fc^*^=kFc[1+0.001xFc^2^λ^3^/sin(2θ)]^-1/4^
Primary atom site location: structure-invariant direct methods	Extinction coefficient: 0.00060 (17)

### 2,4,6-Tribromotoluene (TBT) Special details

**Table:**

Geometry. All esds (except the esd in the dihedral angle between two l.s. planes) are estimated using the full covariance matrix. The cell esds are taken into account individually in the estimation of esds in distances, angles and torsion angles; correlations between esds in cell parameters are only used when they are defined by crystal symmetry. An approximate (isotropic) treatment of cell esds is used for estimating esds involving l.s. planes.

### 2,4,6-Tribromotoluene (TBT) Fractional atomic coordinates and isotropic or equivalent isotropic displacement parameters (Å^2^)

**Table:**

	*x*	*y*	*z*	*U*_iso_*/*U*_eq_	
Br1	0.68701 (2)	1.18762 (9)	0.84471 (2)	0.02351 (10)	
Br2	0.36077 (2)	0.60522 (9)	0.90955 (2)	0.02044 (10)	
Br3	0.35213 (2)	0.68698 (9)	0.55023 (2)	0.02088 (10)	
C1	0.5156 (2)	0.9306 (8)	0.7029 (2)	0.0151 (7)	
C2	0.5592 (2)	0.9823 (8)	0.7965 (2)	0.0165 (7)	
C3	0.5158 (2)	0.8920 (8)	0.8588 (2)	0.0167 (7)	
H3	0.548214	0.934507	0.921902	0.020*	
C4	0.4234 (2)	0.7369 (8)	0.8266 (2)	0.0154 (7)	
C5	0.3755 (2)	0.6790 (8)	0.7351 (2)	0.0148 (7)	
H5	0.312062	0.574516	0.713540	0.018*	
C6	0.4217 (2)	0.7759 (8)	0.6756 (2)	0.0156 (7)	
C7	0.5638 (2)	1.0280 (9)	0.6357 (2)	0.0219 (8)	
H7A	0.576650	0.826805	0.605878	0.033*	
H7B	0.626764	1.142988	0.667500	0.033*	
H7C	0.519404	1.177907	0.589887	0.033*	

### 2,4,6-Tribromotoluene (TBT) Atomic displacement parameters (Å^2^)

**Table:**

	*U*^11^	*U*^22^	*U*^33^	*U*^12^	*U*^13^	*U*^23^
Br1	0.01425 (15)	0.0243 (2)	0.02664 (18)	−0.00447 (13)	0.00050 (13)	0.00205 (15)
Br2	0.02155 (16)	0.0246 (2)	0.01674 (16)	−0.00194 (14)	0.00871 (12)	0.00230 (14)
Br3	0.02070 (16)	0.0273 (2)	0.01272 (15)	−0.00059 (14)	0.00346 (12)	−0.00326 (14)
C1	0.0149 (14)	0.0130 (17)	0.0176 (15)	0.0034 (13)	0.0058 (12)	0.0028 (13)
C2	0.0139 (14)	0.0131 (17)	0.0191 (15)	−0.0009 (12)	0.0016 (12)	0.0024 (13)
C3	0.0176 (15)	0.0133 (17)	0.0146 (15)	0.0003 (13)	−0.0002 (12)	−0.0026 (13)
C4	0.0166 (14)	0.0153 (18)	0.0161 (15)	0.0033 (13)	0.0081 (12)	0.0007 (13)
C5	0.0123 (14)	0.0148 (17)	0.0163 (15)	−0.0015 (12)	0.0039 (12)	−0.0013 (13)
C6	0.0173 (14)	0.0134 (17)	0.0133 (14)	0.0030 (13)	0.0017 (12)	−0.0041 (13)
C7	0.0162 (15)	0.029 (2)	0.0213 (17)	0.0023 (14)	0.0074 (13)	0.0042 (15)

### 2,4,6-Tribromotoluene (TBT) Geometric parameters (Å, º)

**Table:**

Br1—C2	1.907 (3)	C3—H3	0.9500
Br2—C4	1.898 (3)	C4—C5	1.377 (4)
Br3—C6	1.903 (3)	C5—C6	1.376 (5)
C1—C2	1.396 (4)	C5—H5	0.9500
C1—C6	1.405 (4)	C7—H7A	0.9800
C1—C7	1.502 (4)	C7—H7B	0.9800
C2—C3	1.379 (5)	C7—H7C	0.9800
C3—C4	1.389 (4)		
			
C2—C1—C6	114.5 (3)	C6—C5—C4	118.5 (3)
C2—C1—C7	123.6 (3)	C6—C5—H5	120.8
C6—C1—C7	121.9 (3)	C4—C5—H5	120.7
C3—C2—C1	124.1 (3)	C5—C6—C1	123.7 (3)
C3—C2—Br1	116.2 (2)	C5—C6—Br3	116.5 (2)
C1—C2—Br1	119.7 (2)	C1—C6—Br3	119.8 (2)
C2—C3—C4	118.0 (3)	C1—C7—H7A	109.5
C2—C3—H3	121.0	C1—C7—H7B	109.5
C4—C3—H3	121.0	H7A—C7—H7B	109.5
C5—C4—C3	121.2 (3)	C1—C7—H7C	109.5
C5—C4—Br2	119.1 (2)	H7A—C7—H7C	109.5
C3—C4—Br2	119.7 (2)	H7B—C7—H7C	109.5
			
C6—C1—C2—C3	0.0 (5)	C3—C4—C5—C6	0.5 (5)
C7—C1—C2—C3	−179.6 (3)	Br2—C4—C5—C6	179.9 (2)
C6—C1—C2—Br1	179.7 (2)	C4—C5—C6—C1	0.2 (5)
C7—C1—C2—Br1	0.1 (5)	C4—C5—C6—Br3	179.7 (2)
C1—C2—C3—C4	0.7 (5)	C2—C1—C6—C5	−0.5 (5)
Br1—C2—C3—C4	−179.0 (2)	C7—C1—C6—C5	179.2 (3)
C2—C3—C4—C5	−0.9 (5)	C2—C1—C6—Br3	−179.9 (2)
C2—C3—C4—Br2	179.7 (2)	C7—C1—C6—Br3	−0.3 (4)
